# The gap-free genome of *Forsythia suspensa* illuminates the intricate landscape of centromeres

**DOI:** 10.1093/hr/uhae185

**Published:** 2024-07-10

**Authors:** Jian Cui, Congle Zhu, Lisha Shen, Congyang Yi, Rong Wu, Xiaoyang Sun, Fangpu Han, Yong Li, Yang Liu

**Affiliations:** School of Architecture & Built Environment, The University of Adelaide, Adelaide, 5005, Australia; Key Laboratory of Seed Innovation, Institute of Genetics and Developmental Biology, Chinese Academy of Sciences, Beijing, 100101, China; Key Laboratory of Seed Innovation, Institute of Genetics and Developmental Biology, Chinese Academy of Sciences, Beijing, 100101, China; Key Laboratory of Seed Innovation, Institute of Genetics and Developmental Biology, Chinese Academy of Sciences, Beijing, 100101, China; College of Life Science and Technology, Inner Mongolia Normal University, Hohhot, 010022, China; College of Grassland Science, Qingdao Agricultural University, Qingdao, 266109, China; Key Laboratory of Seed Innovation, Institute of Genetics and Developmental Biology, Chinese Academy of Sciences, Beijing, 100101, China; College of Life Science and Technology, Inner Mongolia Normal University, Hohhot, 010022, China; Key Laboratory of Seed Innovation, Institute of Genetics and Developmental Biology, Chinese Academy of Sciences, Beijing, 100101, China

## Abstract

*Forsythia suspensa*, commonly known as weeping forsythia, holds significance in traditional medicine and horticulture. Despite its ecological and cultural importance, the existing reference genome presents challenges with duplications and gaps, hindering in-depth genomic analyses. Here, we present a Telomere-to-Telomere (T2T) assembly of the *F. suspensa* genome, integrating Oxford Nanopore Technologies (ONT) ultra-long, Hi-C datasets, and high-fidelity (HiFi) sequencing data. The T2T reference genome (Fsus-CHAU) consists of 14 chromosomes, totaling 688.79 Mb, and encompasses 33 932 predicted protein-coding genes. Additionally, we characterize functional centromeres in the *F. suspensa* genome by developing a specific CENH3 antibody. We demonstrate that centromeric regions in *F. suspensa* exhibit a diverse array of satellites, showcasing distinctive types with unconventional lengths across various chromosomes. This discovery offers implications for the adaptability of CENH3 and the potential influence on centromere dynamics. Furthermore, after assessing the insertion time of full-length LTRs within centromeric regions, we found that they are older compared to those across the entire genome, contrasting with observations in other species where centromeric retrotransposons are typically young. We hypothesize that asexual reproduction may impact retrotransposon dynamics, influencing centromere evolution. In conclusion, our T2T assembly of the *F. suspensa* genome, accompanied by detailed genomic annotations and centromere analysis, significantly enhances *F. suspensa* potential as a subject of study in fields ranging from ecology and horticulture to traditional medicine.

## Introduction

*Forsythia suspensa*, commonly known as Forsythia, is a prominent deciduous shrub within the Oleaceae family, typically attaining heights of 1.5 to 3.0 meters. Its early spring bloom, featuring abundant, golden, and fragrant flowers, makes it a highly sought-after ornamental shrub for enhancing landscapes. *F. suspensa*’s ecological attributes include its ability to retain soil moisture and its remarkable resistance to cold and drought, which has led to its frequent use for erosion control in hilly terrains and along roadsides. Furthermore, *F. suspensa* holds significant medicinal value, with its fruit extracts being a key component in many traditional Chinese remedies for the common cold. Given its significance in horticulture, ecology, and traditional medicine, Forsythia has gained increasing attention in recent years [1]. The *Forsythia* genus, belonging to Tribe Forsythieae (Oleaceae), includes 10 recognized species and is diploid, with a chromosome number of 2n = 28. *Forsythia likiangensis* and *Forsythia giraldiana* represent the basal lineages, followed by *Forsythia europaea*. These three species are characterized by minutely serrate or entire leaf margins. The remaining species, primarily distributed in East Asia, form two major clades. One clade includes *Forsythia ovata*, *Forsythia velutina*, and *Forsythia japonica*, which are morphologically supported by broadly ovate leaves. The other clade consists of *F. suspensa*, *Forsythia saxatilis*, *Forsythia viridissima*, and *Forsythia koreana*, characterized by lanceolate leaves, except for *F. suspensa*, which has broad ovate leaves [2].

Although a previous study provided a draft genome of weeping forsythia (*F. suspensa*), it did not achieve assembly at the chromosomal level [3]. Building on this, a recent chromosome-scale genomic assembly of weeping forsythia was completed, with a total of 712.9 Mb genomic sequences organized into 14 pseudo-chromosomes [4]. Despite these advances in *F. suspensa* genome research, significant challenges remain. The presence of numerous unassembled or unplaced sequences poses a major obstacle to comprehensive genomic investigations. These challenges emphasize the need for further research and improved genome assembly to fully unlock *F. suspensa*’s potential as a subject of study.

Centromeres serve as crucial genomic structures essential for ensuring the precise segregation of chromosomes during cell division [5]. Two key components are particularly essential in understanding centromere structure and function: CENH3 and centromeric repetitive sequences. CENH3, alternatively referred to as CENP-A in humans, represents a specialized histone H3 variant pivotal for defining centromere identity. Epigenetically labeled, it is uniquely positioned within the centromeric domains of chromosomes. CENH3 assumes a critical function in enlisting additional centromeric proteins and instigating the assembly of the kinetochore, a sophisticated protein complex responsible for interfacing with spindle microtubules during cellular division [6]. Centromeric repetitive sequences, on the other hand, constitute a significant portion of centromere DNA. These repetitive sequences are typically characterized by satellite DNA, which are highly divergent among different species. In plants, centromeres typically encompass numerous copies of simple tandem repeat DNA sequences, organized into extended arrays spanning several megabases in length. These arrays are periodically interrupted by the insertion of long-terminal repeat retrotransposons [7]. The distinctive structural organization of plant centromeres is essential for their proper function and contributes significantly to the precise segregation of chromosomes during cell division [8].

Recent advancements in ultra-long DNA sequencing technologies and the refinement of advanced computational tools have revolutionized the analysis of centromere structure and function. One significant outcome of these advancements is the successful assembly of gap-free reference genomes for several plant species [9–16]. These reference genomes have unveiled the previously concealed complexities and variations within centromeric regions. In Arabidopsis, the processes of satellite homogenization and retrotransposon invasions have been identified as crucial factors in the continuous evolution of centromeres [17]. Furthermore, investigations into Arabidopsis populations have uncovered the existence of centromere diversity and have highlighted the dynamic nature of centromere evolution, driven by rapid cycles of transposon invasion and purging, which are in turn influenced by satellite homogenization [18]. Additionally, during the evolutionary journey of the soybean genome, a remarkable occurrence has been the extensive repositioning of centromeres [19]. This phenomenon of centromere repositioning showcases the genome’s remarkable adaptability in response to evolutionary pressures and genetic changes. It emphasizes the dynamic nature of centromeres and their crucial role in maintaining genome stability. As a result of incomplete assembly in the repetitive regions of previously published *F. suspensa* reference genomes [4], our understanding of the comprehensive size and structural aspects of centromeres in *F. suspensa* remains limited.

In this research, we introduced a gap-free genome assembly of *F. suspensa* through the integration of Oxford Nanopore Technology (ONT) sequencing, PacBio HiFi, and high-throughput chromosome conformation capture (Hi-C) technologies. Our latest assembly, denoted as Fsus-CHAU, surpasses the previously published *F. suspensa* genome in terms of continuity and completeness. This now enables a comprehensive analysis of centromere architecture and evolution on a global scale.

## Results

### A comprehensive, gap-free telomere-to-telomere (T2T) reference genome for *F. suspensa*

**Figure 1:**
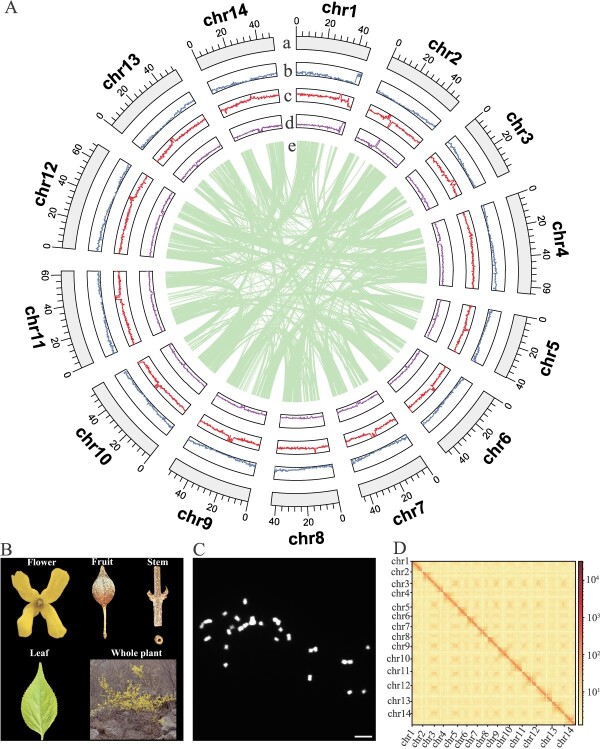
Summary of the Fsus-CHAU genome and its associated phenotypic characteristics. **A** Circos plot. Starting from the outermost to the innermost ring: (a) chromosome, (b) gene number density, (c) LTR density, (d) GC content, and (e) syntenic block. **B** The phenotypes of different tissues, including folwer, fruit, stem, leaf, and the whole plant in *Forsythia suspensa*. **C** The karyotype analysis of 14 chromosome pairs in *F. suspensa*. **D** Chromatin interaction map generated by Hi-C for the Fsus-CHAU genome.

*F. suspensa* was selected for sequencing due to its multifaceted significance in medicine, environmental conservation, and landscape enhancement (Fig. 1B). Through *k*-mer analysis of Illumina reads, we conducted a thorough assessment of the *F. suspensa* genome, yielding a *k*-mer value of 19. This analysis led to an estimated *F. suspensa* genome size of approximately 734.07 Mb, with a calculated heterozygosity level of 1.47% (Table S1, see online supplementary material).

In order to attain a genome assembly of superior quality for *F. suspensa*, we embarked on a comprehensive sequencing endeavor by employing a variety of state-of-the-art technologies. Our approach involved the integration of 58 Gb (~81 × coverage) PacBio HiFi reads, 21 Gb (~30 × coverage) of ONT ultra-long reads, and 78 Gb (~109 × coverage) of Hi-C data (Tables S2–S4, see online supplementary material). After obtaining reads from both HiFi and ONT ultra-long sequencing, primary assembly was conducted, resulting in three sets of primary contig genomes: hifiasm-hifi-ont, which was a hybrid assembly using both HiFi and ONT ultra-long sequencing data; hifiasm-hifi, created using only HiFi sequencing data; and nextdenovo-ont, which utilized ONT ultra-long sequencing data with the NextDenovo software. Following a comprehensive evaluation of each assembly version, the contig-level hifiasm-hifi genome with the highest quality was selected as the T2T genome backbone (Table S5, see online supplementary material). Subsequent analyses, such as removal of assembly contaminants, Hi-C integration, assembly correction, telomere repair, gap filling, and other operations were all based on this version. After Hi-C assembly and manual adjustments, a total of 720 688 960 base pairs of contig sequences were mapped to the 14 chromosomes of the hifiasm-hifi assembly version (Table S6, see online supplementary material), consistent with our karyotype analysis of 14 chromosome pairs in *F. suspensa* (Fig. 1C). However, six gaps remained in the assembly. To address this, we employed ONT ultra-long reads to map onto the initial assembly, filling in these remaining gaps. We found the aligned position spanned across both ends of the six gaps, and thus selected the optimal alignment region from the longest aligned length region for gap filling. This data was then used to replace the sequence in the genome that encompassed the gap region (Fig. S1, see online supplementary material). This comprehensive approach culminated in the creation of a gap-free genome, aptly named Fsus-CHAU. This exceptional genome comprised 14 chromosomes, totaling 688.79 Mb in cumulative length, with an N50 length of 48.48 Mb (Table 1). In the final step of our analysis, we conducted a targeted search using a telomere repeat sequence (5′-CCCTAAA/TTTAGGG-3′). This effort successfully identified 28 telomeres. The upstream copy number of 5′-CCCTAAA ranged from 1117 copies on chromosome 6 to 2363 copies on chromosome 7, and the downstream copy number of 5′-TTTAGGG ranged from 1346 copies on chromosome 7 to 2769 copies on chromosome 9 (Table S7, see online supplementary material). Ultimately, this resulted in the generation of 14 T2T pseudomolecules within the *F. suspensa* assemblies (Fig. 1A).

**Table 1:** Global statistics of Fsus-CHAU genome assembly and annotation.

Assembly	Fsus-CHAU	Annotation	Fsus-CHAU
Genome size (Mb)	688.79	Gene number	33 932
Number of contigs	155	Genome BUSCO (%)	98.6
Contig N50 (Mb)	48.48	Average gene length (bp)	5279
Number of contigs (length ≥2 kb)	14	Number of non-coding RNAs	8398
Number of gap-free chromosomes	14	TE percentage (%)	62
Number of telomeres	28	Gypsy family percentage (%)	14
Number of definite centromeres	14	Copia family percentage (%)	13

**Figure 2:**
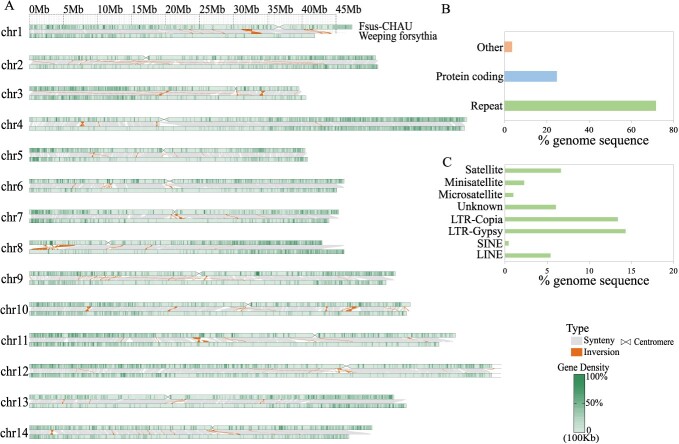
Collinearity between Fsus-CHAU and weeping forsythia. **A** Structural variations between the Fsus-CHAU and weeping forsythia genome. **B** Summary of genomic annotation: the ‘Other’ genomic composition encompasses non-protein-coding genes, unclassified repeat regions, and genomic segments that defy annotation as either genes or repeats. **C** Proportion of different repeat types in the Fsus-CHAU genome.

The precision and comprehensiveness of the *F. suspensa* genome assembly underwent extensive validation through various methods. Initially, the Hi-C heatmap clearly indicated a strong uniformity among all chromosomes, confirming the accurate organization and alignment of contigs in the new assembly (Fig. 1D). Subsequently, the assembly’s accuracy was further bolstered by the observation that over 99% of PacBio HiFi reads were successfully aligned to the new assembly (Table S8, see online supplementary material). Lastly, the completeness of the assembly was evaluated using the Benchmarking Universal Single-Copy Orthologs (BUSCO) analysis, achieving an impressive 98.6% completion rate (1591 out of 1614 BUSCOs) (Table 1; Table S9, see online supplementary material). Our gap-free Fsus-CHAU genome demonstrates superior contiguity and completeness compared to previously reported assembly (Fig. 2A). Specifically, our assembly boasts a contig N50 of 48.48 Mb, significantly higher than the previous weeping forsythia genome’s contig N50 of 7.3 Mb. Previous reference genomes contained hundreds or thousands of gaps [4]. However, our assembly has added 8.91 Mb of new sequences and effectively filled in all existing gaps (Fig. 2A, Table 1).

### Genome annotation

In the process of annotating the gene models of *F. suspensa* genome, we employed a comprehensive approach that combined multiple prediction methods, homology-based analyses, and transcriptome data. This rigorous annotation pipeline allowed us to achieve a thorough and accurate gene annotation (Fig. S2, see online supplementary material). Our analysis identified a total of 33 932 gene models in the *F. suspensa* genome (Table 1; Table S10, see online supplementary material). Notably, these gene models captured 98.0% of the BUSCO 1614 reference gene set, further attesting to the quality of our annotation. The average gene length was estimated to be 5279 bp, with an average of 4.77 exons per gene (Table S11, see online supplementary material). Additionally, 20 242 genes were supported by transcriptome data, accounting for 59.65% of the total gene models. In parallel with protein-coding genes, our analysis also predicted 8398 non-coding RNAs within the *F. suspensa* genome. This encompassed 190 microRNAs, 716 transfer RNAs, 4919 ribosomal RNAs, and 2573 small nuclear RNAs, contributing to a comprehensive and detailed gene annotation (Table 1; Table S12, see online supplementary material).

For a comprehensive understanding of the repetitive landscape in the *F. suspensa* genome, we leveraged a combination of *de novo* and homoeology-based methods. Ultimately, we identified that approximately 72% of our gap-free genome is comprised of repetitive sequences (Fig. 2B; Tables S13 and S14, see online supplementary material). Notably, the majority of these repeats were attributed to transposable elements (TEs), encompassing 62% of the genome and totaling 450 664 727 bp in length. The TE category was predominantly characterized by the long terminal repeat (LTR) type, with 14% of these elements belonging to the Gypsy family and 13% to the Copia family (Fig. 2C, Table 1; Table S13, see online supplementary material).

**Figure 3:**
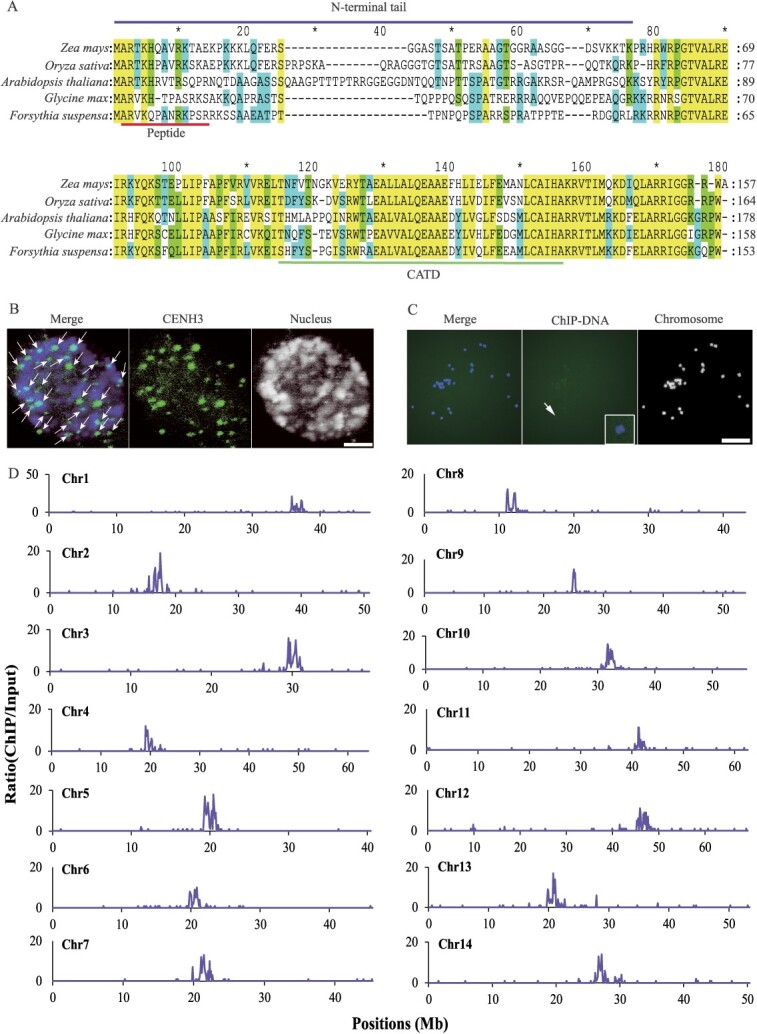
Genomic mapping of functional centromeres in *Forsythia suspensa*. **A** Multiple alignments of CENH3 homologs from different plant lineages including *Zea mays*, *Oryza sativa*, *Arabidopsis thaliana*, *Glycine max*, and *F. suspensa*. The peptide used to generate the anti-CENH3 antibody, with the N-terminal tail and the conserved CENP-A Targeting Domain (CATD) marked on the sequence. **B** Immunostaining of nucleus in the *F. suspensa* using the anti-CENH3 antibody. Arrows indicate CENH3 signals. Bar = 10 μm. **C** FISH assay using CENH3-ChIPed DNA as probes. The boxed insets show high-magnification image of the chromosome indicated by the arrow. Chromosomes are stained with DAPI. Bar = 10 μm. **D** This schematic overview illustrates the results of the CENH3 ChIP-seq assay in *F. suspensa*. The mapped reads density was plotted in 100-kb windows along the 14 chromosomes. The x-axis shows the positions on each chromosome. The y-axis represents ChIP/input ratio of the normalized density of uniquely mapped reads on each position, which was calculated based on the ratio of reads per million/100.

### Genomic mapping of functional centromeres in *F. suspensa*

While utilizing centromere-specific repeats provides valuable resources, the precise determination of centromere positions is a concern, particularly in light of the extensive centromere repositioning phenomena observed across various species during evolution [20–24]. To accurately locate the centromeres within the *F. suspensa* genome, we developed a highly specific antibody against the single copy of the *CENH3* gene (FsCENH3) identified in the genome (Fig. 3A). This antibody, crafted from a peptide that corresponds to the N-terminal segment of the FsCENH3 sequence, displayed the remarkable ability to precisely target all functional *F. suspensa* centromeres (Fig. 3B). We next harnessed chromatin immunoprecipitation sequencing (ChIP-seq) with the purpose-designed anti-CENH3 antibody for *F. suspensa*. The application of fluorescence in situ hybridization (FISH) with ChIPed DNA as the probe revealed distinct, strong signals at the centromeres, suggesting that we successfully captured the majority of genuine centromeric DNA in our samples (Fig. 3C). The DNA fragments successfully immunoprecipitated were subsequently subjected to high-throughput sequencing on the NovaSeq platform. This sequencing data was analysed alongside control DNA samples, carefully extracted from chromatin preparations before commencing the ChIP procedure. Our sequencing analysis generated an average of 16 million reads, achieving a coverage of 6× (Table S15, see online supplementary material). After stringent filtering, the refined reads were accurately aligned to the Fsus-CHAU genome. This unveiled a notable concentration of CENH3 peaks within the centromeric domains spanning all 14 chromosomes of *F. suspensa* (Fig. 3D). The core region of CENH3 binding in *F. suspensa* centromeres spans a total of 12.9 Mb, ranging from 0.4 to 1.5 Mb in size, which accounts for 1.87% of the 688.79 Mb *F. suspensa* genome, with a mean centromere size of approximately 0.9 Mb (Table 2). We also employed the CentroMiner module within the quarTeT software to identify centromere positions [25]. The results showed that quarTeT accurately identified centromere locations, consistent with functional centromere positions identified by CENH3-ChIP-seq (Fig. S3, see online supplementary material). In summary, we have successfully identified and characterized the functional centromeres in the *F. suspensa* genome.

**Table 2:** Locations of functional CENH3-binding sites in *Forsythia suspensa*.

Chr	Chr size (Mb)	Cen location (Mb)	Cen size (Mb)	CentFs arrays (Mb)	Chr	Chr size (Mb)	Cen location (Mb)	Cen size (Mb)	CentFs arrays (Mb)
1	47	35.9–37.3	1.4	1.3	8	43	11.2–12.0	0.8	1.0
2	51	16.7–17.5	0.8	3.0	9	54	24.4–25.4	1.0	3.7
3	40	30.1–30.5	0.4	4.9	10	56	31.6–32.6	1.0	3.5
4	64	19.0–20.5	1.5	3.2	11	62	41.5–42.3	0.8	1.0
5	40	19.4–20.0	0.6	1.5	12	69	45.8–47.2	1.4	4.0
6	46	19.8–21.3	1.5	1.5	13	53	19.9–20.7	0.8	2.8
7	45	21.1–21.6	0.5	4.0	14	50	26.7–27.1	0.4	4.0
		Cen, centromere; Chr, chromosome; CentFs, *F. suspensa* centromeric satellites.							

### The centromeres contain rich and diverse satellite sequences in *F. suspensa*

**Figure 4:**
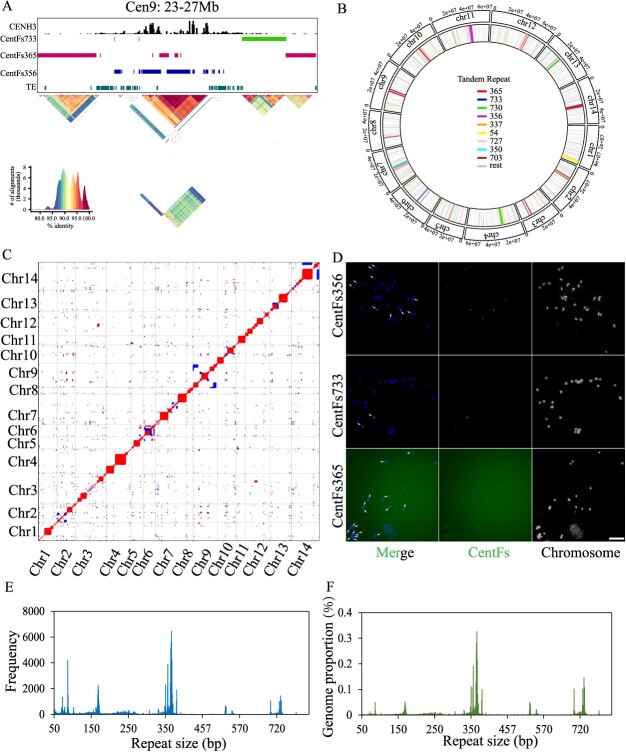
Characterization of the diverse centromeric satellites in *Forsythia suspensa*. **A** Characteristics of centromeres within *F. suspensa* genome assemblies. The enrichment of CENH3 [log_2_(ChIP/Input)] depicted across centromere 9. **B** A circos plot depicting tandem repeats identified by TRASH in *de novo* mode within the Fsus-CHAU genome assembly. **C** Dot plots comparing the centromeres using a search window of 100 bp. Forward- and reverse-strand similarity are indicated by different points. **D** FISH signals of CentFs356, CentFs365, and CentFs733 in *F. suspensa*. Bar = 10 μm. **E** A histogram illustrating the lengths (bp) of tandem repeats identified in the Fsus-CHAU genome assembly by TRASH. **F** Proportion of the genome occupied by different tandem repeats.

To unravel the sequence composition of *F. suspensa* centromeres, we embarked on a comprehensive quest to characterize centromere-specific repetitive elements within the *F. suspensa* genome. Our approach began with utilizing RepeatExplorer to scrutinize and delineate repeat clusters. Among the clusters examined, 20 showed a CENH3-ChIP/input ratio surpassing 7, with their genomic representation exceeding 0.05% (Fig. S4A, see online supplementary material). Through the use of the Basic Local Alignment Search Tool (BLAST), we pinpointed four clusters that were specifically associated with the centromeres (Fig. S4B–E, see online supplementary material). All four clusters were identified as satellite repeats arranged in tandem, each with a distinct unit size: 356, 365, 530, and 732 base pairs, respectively (Fig. S4B–E, see online supplementary material). These were named CentFs356, CentFs365, CentFs530, and CentFs732 according to their respective lengths. We also utilized the *de novo* mode of the Tandem Repeat Annotation and Structural Hierarchy (TRASH) tool to investigate tandem repeats. This tool not only validated the existence of four distinct satellite populations as identified but also uncovered novel centromere-specific satellites. These satellites, particularly those with lengths around 360 bp and 730 bp, emerged as the predominant types (Fig. 4E and F). Interestingly, the primary satellites of centromeres varied across different chromosomes (Fig. 4B). For example, while CentFs54 was prevalent in centromere 1, CentFs727 was dominant in centromere 12 (Fig. 4B). Moreover, most centromeres exhibited a blend of satellites; for instance, centromere 4 was composed mainly of CentFs356 and CentFs365, whereas centromere 9 comprised primarily CentFs356, CentFs365, and CentFs733 (Fig. 4A). We selected four centromeric satellites and designed specific oligo probes. Subsequent FISH analyses unveiled the localization of all centromeric satellites to their respective centromeric regions (Fig. 4D; Fig. S5, see online supplementary material). Notably, variations in signal intensities were observed, suggesting differences in abundance among distinct centromeric regions (Fig. 4D). Additionally, dot plot analysis unveiled significant discrepancies in the genomic landscape of centromeres, underscoring the unique sequence and structural features of each centromere in *F. suspensa* (Fig. 4C). These findings highlight the intricate and diverse organization of centromeric satellites in *F. suspensa*, suggesting a dynamic evolutionary process with potentially multifaceted functions.

CENH3 nucleosomes exhibit a distinctive highly phased arrangement at specific sites along centromere satellites, a crucial aspect believed to contribute to proper centromere function [26–28]. To explore whether CENH3 nucleosomes in *F. suspensa* bind randomly to centromeric satellite sequences of large monomer size or target specific sites. Employing a methodology akin to centromere analyses in humans and plants [26, 29], we combined CENH3-ChIP reads, indicative of CENH3-bound nucleosomes, with input reads, representing bulk nucleosome fragments. These reads were subsequently aligned to consensus satellite sequences of trimer repeats (comprising three copies of a consensus monomer). The distribution of fragment midpoints was then utilized to determine the positioning of CENH3 or canonical nucleosomes. From the CENH3-ChIP reads, we observed five distinct binding peaks in the trimeric CentFs356 (Fig. S6A, see online supplementary material), and two prominent binding peaks in the trimeric CentFs365 (Fig. S6B, see online supplementary material), CentFs530 (Fig. S6C, see online supplementary material), and CentFs732 (Fig. S6D, see online supplementary material) consensus sequence. This suggests a phased arrangement of CENH3 nucleosomes on *F. suspensa* centromeric satellite sequences. Notably, the positions of input nucleosomes on the monomer mirrored those of the CENH3 nucleosomes (Fig. S6A–D, see online supplementary material), implying that a significant portion of repeats in CentFs356, CentFs365, CentFs530, and CentFs732 were enfolded around the CENH3 nucleosomes.

### Asexual reproduction in *F. suspensa* may have impacted the dynamics of retrotransposon invasions, thereby driving the evolution of centromeres

**Figure 5:**
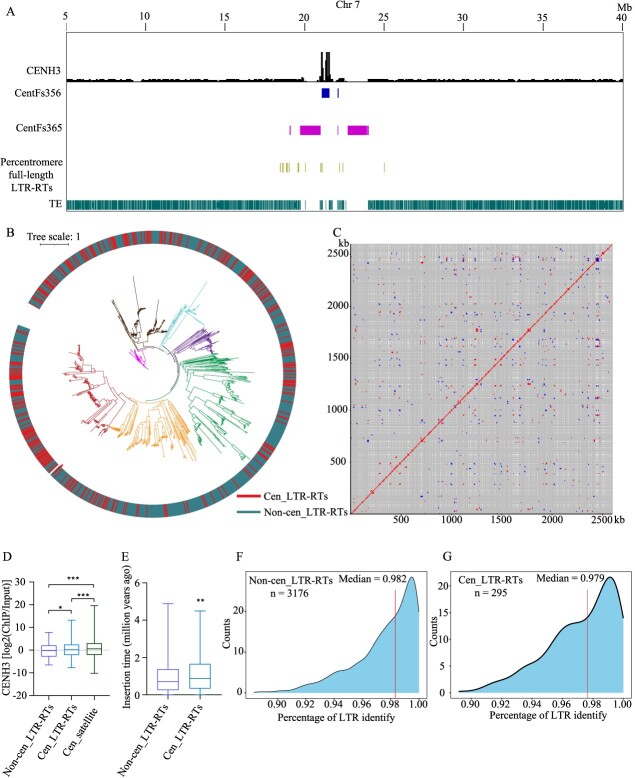
The LTR-RTs in the centromeres of *Forsythia suspensa*. **A** The distribution of full-length LTR-RTs, fragmented LTRs, and centromeric satellites within the extended 20 Mb region surrounding the centromere of Chromosome 7. **B** Phylogenetic tree of full-length LTR-RTs from the Fsus-CHAU genome, with branches differentiated by their locations within and outside the centromeres. **C** Dot plot of centromeric full-length LTR-RTs from the Fsus-CHAU genome using a 100-bp search window. **D** CENH3 enrichment level [log2(ChIP/Input)] around CentFs satellites, centromeric full-length LTR-RTs (*n* = 295) and non-centromeric full-length LTR-RTs (*n* = 3176). **E** Comparison of the insertion timing between full-length LTR-RTs located within centromeres (Cen_LTR-RTs) and those outside of centromeres (Non-cen_LTR-RTs). **F** Non-centromeric full-length LTR-RTs (Non-cen_LTR-RTs): the histogram displays the distribution of LTR identity percentages for non-cen_LTR-RTs, with a sample size of 3176 elements. The median LTR identity is 0.982, as indicated by the vertical red line. The x-axis represents the percentage of LTR identity, while the y-axis shows the count of LTR-RTs within each identity range. **G** Centromeric full-length LTR-RTs (Cen_LTR-RTs): the histogram shows the distribution of LTR identity percentages for centromeric LTR-RTs, with a sample size of 295 elements. The median LTR identity is 0.979, marked by the vertical red line. The x-axis represents the percentage of LTR identity, and the y-axis indicates the count of LTR-RTs within each identity range.

We observed a notable disruption of satellite arrays attributed to retrotransposons (Fig. 5A). We meticulously annotated the full-length LTR-RTs within the genome of Fsus-CHAU. Our analysis revealed a total of 3471 full-length LTR-RTs, with 295 located specifically within the centromeric regions (Fig. 5F and G). Notably, we detected instances of fragmented and nested LTR insertions within these centromeres (Fig. 5A). A detailed examination, represented through dot plots, showcased substantial sequence divergence among centromeric full-length LTR-RTs (Fig. 5C), leading to their classification into seven distinct clades (Fig. 5B). Interestingly, these LTR-RTs clustered alongside those found outside the centromeres, indicating subsequent duplications post-integration within the centromeres (Fig. 5B). Furthermore, our investigation unveiled a higher enrichment level of CENH3 on the satellite arrays compared to the centromeric retrotransposons (Fig. 5D). This observation suggests that the repetitive nature or chromatin structure of satellite arrays potentially renders them more favorable binding sites for CENH3.

In assessing the insertion time of full-length LTRs within centromeric regions, we discovered that LTRs within the centromeres are older than those present across the entire genome (Fig. 5E). LTR identity can indicate the age of centromeric retrotransposons because when a retrotransposon is first inserted, its LTR sequences are identical. Over time, mutations accumulate independently in each LTR, leading to decreased sequence identity. Thus, lower LTR identity suggests a longer time since insertion, indicating an older age [30]. We analysed LTR identity between centromeric and non-centromeric regions. On average, LTR identities in non-centromeric regions were notably higher than those within centromeres, indicating the comparatively older age of centromeric retrotransposons (Fig. 5F and G). This finding contrasts with reports from other species, where very young CRM retrotransposons were observed in centromeres [31–34]. Recent studies have suggested a decrease in transposable element loads under asexual reproduction [35]. Given that *F. suspensa* predominantly reproduces asexually, primarily through methods like vegetative propagation rather than sexual reproduction, this raises intriguing possibilities that asexual reproduction could influence the dynamics of retrotransposon invasions, which in turn may have shaped the evolution of centromeres.

## Discussion

The *F. suspensa* genome has long been a subject of interest, with recent advances in research shedding light on its complexities and challenges. *F. suspensa* faces obstacles arising from incomplete assembly in repetitive regions, hindering a comprehensive understanding of its genomic landscape. In response to these challenges, we undertook an extensive genome sequencing initiative, employing state-of-the-art technologies such as ONT sequencing, PacBio HiFi, and Hi-C. The resulting T2T assembly, denoted as Fsus-CHAU, represents a significant leap forward, surpassing previous genomes in continuity and completeness. Fsus-CHAU consists of 14 chromosomes with a total cumulative length of 688.79 Mb and an N50 length of 48.48 Mb. Similar to the utilization of reference genomes in other plant communities, Fsus-CHAU serves as a valuable resource for in-depth exploration of *F. suspensa*’s genomic intricacies.

Centromeres play a crucial role in ensuring accurate chromosome segregation and genetic material distribution during cell division. Unlike sequences found on chromosome arms, centromeres are distinguished by a dense concentration of repetitive elements specific to the centromeric region. Researchers frequently rely on these centromere-specific repetitive elements to pinpoint functional centromeres in T2T genomes. However, this approach encounters a significant challenge: centromeric satellite arrays are often larger than the actual functional centromeric regions. CENH3 immunolocalization provides a direct method to pinpoint functional centromeres. This methodological robustness is particularly valuable in studies involving species prone to centromere dynamics and genomic rearrangements. A key milestone in our study was the first-time identification of the *CENH3* within the Forsythia genus. Furthermore, our study advanced beyond gene identification by taking a proactive step in characterizing functional centromeres in *F. suspensa*. This involved the development of a highly specific CENH3 antibody. The application of this antibody allowed us to precisely target and identify functional centromeric regions in *F. suspensa*. We found the average size of the core region of CENH3 binding in *F. suspensa* centromeres is 0.9 Mb, markedly smaller than the size of the *F. suspensa* centromeric satellite arrays (Table 2). These observations align with recent analyses conducted in soybean and Arabidopsis [17, 19]. This common pattern implies that the proportional relationship between the size of CENH3-enriched regions and the broader satellite arrays could serve as a shared organizational characteristic in centromeres across various plant species.

In our study, we investigated the centromere structure of *F. suspensa* and found that its centromeres are characterized by a high density of satellite DNA arrays, which are tandemly repeated sequences specific to the centromeric region. A small portion of LTR-RTs interspersed within these satellite arrays potentially influence centromere function and stability. This centromere structure is similar to that observed in grape [36, 37], soybean [19], maize [38], and Arabidopsis [39], which also have satellite-rich centromeres. In contrast, species such as wheat [32], oats [40], and rye [41] have centromeres that are rich in retrotransposons, lacking extensive satellite DNA arrays and instead being dominated by retrotransposon sequences. The differences between satellite-rich and retrotransposon-rich centromeres highlight the diversity of centromere evolution and its implications for genome dynamics. Satellite-rich centromeres, despite their disruptions by retrotransposons, maintain a high degree of sequence specificity and structural integrity essential for proper chromosome segregation. The interplay between satellite DNA and retrotransposons in these centromeres suggests a balance between maintaining centromere stability and allowing for evolutionary flexibility. On the other hand, retrotransposon-rich centromeres represent a more fluid and dynamic centromere architecture. The dominance of retrotransposons can lead to rapid centromere evolution and adaptation, potentially offering advantages in rapidly changing environments or under selective pressures. The identified satellites in *F. suspensa* exhibit a notable departure from the typical monomeric lengths associated with well-established centromeric repeats. Established repeats often consist of monomers ranging from 150 to 180 base pairs, exemplified by well-known sequences such as pAL1 in *Arabidopsis* [39] and CentC in maize [38]. Such monomeric lengths are crucial because they can accommodate a single CENH3 nucleosome, which in turn aids in the assembly of CENH3 nucleosome arrays with a specific arrangement throughout the chromosome, essential for the establishment and stability of centromeres. Contrastingly, the newly identified centromeric satellites in *F. suspensa* showcase a wide range of monomeric lengths, spanning from 300 base pairs to 700 base pairs. This variation, often characterized as ‘odd’ repeats, has been observed to be limited to specific centromeres and not universally present across all centromeres. The unconventional lengths of these repeats suggest a dynamic process of amplification and adaptation. This dynamic nature raises intriguing questions about the functional implications of such satellite structures in centromere biology.

Based on our findings, the age distribution and identity of LTRs within centromeric regions present intriguing insights into the dynamics of retrotransposon invasions and their impact on centromere evolution in *F. suspensa*. Contrary to observations in other species where young CRM retrotransposons dominate centromeric regions, our study reveals a striking pattern: LTRs within the centromeres of *F. suspensa* are notably older than those across the entire genome (Fig. 5E–G). This departure from the norm prompts a reevaluation of the interplay between asexual reproduction and retrotransposon dynamics. The prevalence of asexual reproduction in *F. suspensa*, primarily through mechanisms such as vegetative propagation, suggests a potential link between reproductive strategy and retrotransposon dynamics. Recent studies have hinted at a reduction in transposable element loads under asexual reproduction conditions [35]. Our findings add weight to this hypothesis by indicating that asexual reproduction might influence the dynamics of retrotransposon invasions, thereby shaping the evolutionary trajectory of centromeres. This departure from the expected pattern underscores the complexity of centromere evolution and highlights the need for a nuanced understanding of the interplay between genomic dynamics and reproductive strategies. Furthermore, our study opens up avenues for exploring the mechanisms underlying retrotransposon regulation and their role in shaping centromere architecture across different species. Future research could delve deeper into the molecular mechanisms mediating retrotransposon dynamics under varying reproductive strategies, shedding light on the broader evolutionary implications for genome organization and stability in diverse taxa.

## Materials and methods

### Plant materials, DNA library construction, and sequencing

The wild *F. suspensa* was cultivated within a greenhouse environment under controlled conditions for 30 days, maintaining a 16-hour light and 8-hour dark cycle at a temperature of 28°C. For Oxford Nanopore Ultra-long sequencing, the libraries were constructed according to standard protocols and sequencing was conducted using a PromethION sequencer by Oxford Nanopore Technologies (Oxford, Oxfordshire, UK). For PacBio HiFi sequencing, libraries were prepared using the Pacific Biosciences SMRT bell express template prep kit 2.0 following the standard protocol. Subsequently, a SMRT cell sequencing library was created, containing approximately 15 kb cut fragments, and sequenced using the PacBio sequel II sequencing platform with 30-hour runs (Pacific Biosciences, Menlo Park, CA, USA). For Hi-C sequencing, we utilized high-quality DNA extracted from the youthful leaves of *F. suspensa* species. Chromatin was appropriately fixed using formaldehyde, and the in situ Hi-C chromosome conformation capture procedure was executed following the DNase-based protocol originally outlined by Ramani [42]. Subsequently, sequencing of the Hi-C libraries was conducted using the Illumina NovaSeq platform in paired-end 150-bp sequence reads (Illumina, San Diego, CA, USA). For Illumina RNA sequencing, the leaf samples were used as input material for the RNA sample preparations. Sequencing libraries were generated using NEBNext® Ultra™ RNA Library Prep Kit for Illumina® (#E7530L, NEB, USA) following the manufacturer’s recommendations, then the libraries were sequenced on an Illumina platform and 150 bp paired-end reads were generated, all of which were conducted at Wuhan Benagen Technology Co., Ltd in Wuhan, China.

### Genome assembly

The genome assembly employed various strategies based on different algorithms, resulting in four primary sets of contig genomes. Consensus reads were filtered using the CCS software with default parameters. High-quality HiFi reads were utilized for assembly through Hifiasm with default parameters, resulting in a draft genome [43]. ONT ultralong sequencing reads were filtered, and only those of high quality were employed for assembly using NextDenovo [44] with default parameters.

Following initial assembly, Racon [45] and Pilon [46] were applied for three rounds of correction and polishing with ONT and Illumina PE reads, yielding the final contigs. Hi-C data were processed using fastp [47] to clean the data, while HiCUP extracted valid interaction pairs to facilitate chromosome-level assembly. Subsequently, ALLHiC [48] and Juicebox [49] clustered, ordered, and oriented the contigs. The Hifiasm-generated assembly served as the backbone genome, with the ONT genome filling gaps in the backbone. The genome underwent further correction with aligned HiFi reads using Winnowmap. Finally, a genomic interaction heatmap for the T2T genome was generated using HiCExplorer [50].

### Genes and transposable elements annotation

For transposable elements annotation, the *de novo* approach was employed using the RepeatModeler software [51] to predict model sequences from the genomic sequence. Simultaneously, the LTR_FINDER software [52] and LTRharvest [53] were used to predict LTR sequences. The LTR_retriever software [54] was then applied to remove redundancy from the results obtained by the two aforementioned methods, resulting in a non-redundant set of LTR sequences. The *de novo* repeat sequence library was created by merging the results of these two *de novo* sequence prediction methods. The teclass software [55] was utilized to redefine the unknown content within this library. Subsequently, the RepBase library and the *de novo* library were merged, and the RepeatMasker software was employed to align and predict repetitive sequences, yielding the de novo + repbase results. Finally, the results of all repetitive predictions were consolidated, and redundant entries were eliminated to yield the final dispersed repetitive sequence set, referred to as Combined TEs.

Gene annotation encompassed a comprehensive approach, integrating *ab initio* predictions, homology-based methods, and RNA-seq data-supported strategies. Two *ab initio* gene prediction tools, Augustus [56] and GlimmerHMM [57], were employed, with BUSCO [58] aiding in the acquisition of training sets for these predictors. Homology-based gene prediction involved the selection of reference species, including *Fraxinus pennsylvanica*, *Olea europaea*, *Jasminum sambac*, and *Osmanthus fragrans* with Exonerate used for the prediction. RNA-seq reads underwent filtration using fastp and were aligned to the assembled genome through hisat2 [59]. Afterward, gene models were predicted using StringTie [60] and TransDecoder. This integrated approach aimed to enhance the accuracy and completeness of gene annotation by leveraging the strengths of multiple prediction methods and supporting evidence from RNA-seq data.

### CENH3-ChIP-seq library construction and data analysis

CENH3-ChIP-seq was conducted as previously described [40]. Approximately 5 g of young leaves from *F. suspensa* were utilized for the ChIP experiment. The libraries underwent sequencing on the Illumina NovaSeq platform to produce paired-end 150-bp sequence reads. The processing and alignment of CENH3-ChIP-Seq raw data were conducted as previously described [19].

### Identifying and characterizing the centromeric repeats

A total of one million randomly selected sequence reads (150 bp) from the input control were analysed using the web-based GALAXY REPEATEXPLORER (https://repeatexplorer-elixir.cerit-sc.cz/galaxy/) with default parameters. This analysis aimed to scrutinize and delineate repeat clusters. The repeats were identified and classified into individual repeat clusters based on their sequence similarity. To estimate the genome proportion of each repeat cluster, we counted the number of reads in each cluster. This step provided us with the genomic representation of each repeat cluster.

Next, to identify the repeats associated with the CENH3-containing chromatin, we conducted a BLAST analysis. The CENH3 ChIP-seq and input reads were separately subjected to BLASTn analysis against the repeat clusters using an E-value threshold of 1e–8. This process enabled us to determine which repeat sequences were enriched in the CENH3 ChIP-seq data compared to the input control. The CENH3-ChIP/input ratio was calculated by comparing the number of aligned reads from the CENH3 ChIP-seq data to the number of aligned reads from the input data for each repeat cluster. This ratio represents the relative enrichment of each repeat cluster in the CENH3 ChIP-seq data. A higher ratio indicates a greater association of that repeat cluster with CENH3-containing chromatin.

### LTR retrotransposon insertion time

Estimating the insertion time of LTR retrotransposons involves analysing the divergence between their two LTR sequences, which accumulate mutations after integration into the host genome. This process utilizes sequence alignment tools to compare the LTR sequences and calculate their differences or percent divergence. By knowing the mutation rate of LTR sequences, typically estimated from known rates in related species, the insertion time can be approximated using the formula T = K/ (2 * r), where K is the number of differences and r is the mutation rate per site per year [30].

## Supplementary Material

Web_Material_uhae185

## Data Availability

The genome sequence data, the genome assembly, and the associated annotation have been deposited at the National Genomics Data Center and can be accessed with the accession code PRJCA023056.

## References

[1] Liu Q, Chen X, Guo L. et al. Comparison of the growth adaptation of six Forthysia species in the quaternary red soil of South China. Res Soil Water Conserv. 2020;27:357–69

[2] Ha YH, Kim C, Choi K. et al. Molecular phylogeny and dating of Forsythieae (Oleaceae) provide insight into the Miocene history of Eurasian temperate shrubs. Front Plant Sci. 2018;9:99 29459880 10.3389/fpls.2018.00099 PMC5807412

[3] Li LF, Cushman SA, He YX. et al. Genome sequencing and population genomics modeling provide insights into the local adaptation of weeping forsythia. Hortic Res. 2020;7:130 32821413 10.1038/s41438-020-00352-7 PMC7395120

[4] Li Y, Wang F, Pei N. et al. The updated weeping forsythia genome reveals the genomic basis for the evolution and the forsythin and forsythoside a biosynthesis. Hortic Plant J. 2023;9:1149–61

[5] Liu C, Huang Y, Guo X. et al. Young retrotransposons and non-B DNA structures promote the establishment of dominant rye centromere in the 1RS.1BL fused centromere. New Phytol. 2024;241:607–22 37897058 10.1111/nph.19359

[6] Zhou J, Liu Y, Guo X. et al. Centromeres: from chromosome biology to biotechnology applications and synthetic genomes in plants. Plant Biotechnol J. 2022;20:2051–63 35722725 10.1111/pbi.13875 PMC9616519

[7] Liu Y, Liu Q, Su H. et al. Genome-wide mapping reveals R-loops associated with centromeric repeats in maize. Genome Res. 2021;31:1409–18 34244230 10.1101/gr.275270.121 PMC8327920

[8] Naish M, Henderson IR. The structure, function, and evolution of plant centromeres. Genome Res. 2024;34:161–78 38485193 10.1101/gr.278409.123 PMC10984392

[9] Zhang A, Kong T, Sun B. et al. A telomere-to-telomere genome assembly of Zhonghuang 13, a widely-grown soybean variety from the original center of Glycine max. Crop J. 2023;12:142–53

[10] Li G, Tang L, He Y. et al. The haplotype-resolved T2T reference genome highlights structural variation underlying agronomic traits of melon. Hortic Res. 2023;10:uhad182 37885818 10.1093/hr/uhad182 PMC10599238

[11] Xu X-D, Zhao RP, Xiao L. et al. Telomere-to-telomere assembly of cassava genome reveals the evolution of cassava and divergence of allelic expression. Hortic Res. 2023;10:uhad200 38023477 10.1093/hr/uhad200 PMC10673656

[12] Pei T, Zhu S, Liao W. et al. Gap-free genome assembly and CYP450 gene family analysis reveal the biosynthesis of anthocyanins in *Scutellaria baicalensis*. Hortic Res. 2023;10:uhad235 38156283 10.1093/hr/uhad235 PMC10753160

[13] Huang H-R, Liu X, Arshad R. et al. Telomere-to-telomere haplotype-resolved reference genome reveals subgenome divergence and disease resistance in triploid Cavendish banana. Hortic Res. 2023;10:uhad153 37701454 10.1093/hr/uhad153 PMC10493638

[14] Ma B, Wang H, Liu J. et al. The gap-free genome of mulberry elucidates the architecture and evolution of polycentric chromosomes. Hortic Res. 2023;10:uhad111 37786730 10.1093/hr/uhad111 PMC10541557

[15] Li B, Yang Q, Yang L. et al. A gap-free reference genome reveals structural variations associated with flowering time in rapeseed (*Brassica napus*). Hortic Res. 2023;10:uhad171 37841499 10.1093/hr/uhad171 PMC10569240

[16] Jia KH, Zhang X, Li LL. et al. Telomere-to-telomere cultivated and wild soybean genome assembly provides insights into evolution and domestication under structural variation. Plant Commun. 2024;100919 38605518 10.1016/j.xplc.2024.100919 PMC11369727

[17] Naish M, Alonge M, Wlodzimierz P. et al. The genetic and epigenetic landscape of the Arabidopsis centromeres. Science. 2021;374:eabi7489 34762468 10.1126/science.abi7489 PMC10164409

[18] Wlodzimierz P, Rabanal FA, Burns R. et al. Cycles of satellite and transposon evolution in Arabidopsis centromeres. Nature. 2023;618:557–65 37198485 10.1038/s41586-023-06062-z

[19] Liu Y, Yi C, Fan C. et al. Pan-centromere reveals widespread centromere repositioning of soybean genomes. Proc Natl Acad Sci USA. 2023;120:e2310177120 37816061 10.1073/pnas.2310177120 PMC10589659

[20] Schubert I . What is behind "centromere repositioning"? Chromosoma. 2018;127:229–34 29705818 10.1007/s00412-018-0672-y

[21] Nasuda S, Hudakova S, Schubert I. et al. Stable barley chromosomes without centromeric repeats. Proc Natl Acad Sci USA. 2005;102:9842–7 15998740 10.1073/pnas.0504235102 PMC1175009

[22] Mandáková T, Hloušková P, Koch MA. et al. Genome evolution in Arabideae was marked by frequent centromere repositioning. Plant Cell. 2020;32:650–65 31919297 10.1105/tpc.19.00557 PMC7054033

[23] Han Y, Zhang Z, Liu C. et al. Centromere repositioning in cucurbit species: implication of the genomic impact from centromere activation and inactivation. Proc Natl Acad Sci USA. 2009;106:14937–41 19706458 10.1073/pnas.0904833106 PMC2736423

[24] Montefalcone G, Tempesta S, Rocchi M. et al. Centromere repositioning. Genome Res. 1999;9:1184–8 10613840 10.1101/gr.9.12.1184 PMC311001

[25] Lin Y, Ye C, Li X. et al. quarTeT: a telomere-to-telomere toolkit for gap-free genome assembly and centromeric repeat identification. Hortic Res. 2023;10:uhad127 37560017 10.1093/hr/uhad127 PMC10407605

[26] Su H, Liu Y, Liu C. et al. Centromere satellite repeats have undergone rapid changes in Polyploid wheat subgenomes. Plant Cell. 2019;31:2035–51 31311836 10.1105/tpc.19.00133 PMC6751130

[27] Yang X, Zhao H, Zhang T. et al. Amplification and adaptation of centromeric repeats in polyploid switchgrass species. New Phytol. 2018;218:1645–57 29577299 10.1111/nph.15098

[28] Ioshikhes I, Hosid S, Pugh BF. Variety of genomic DNA patterns for nucleosome positioning. Genome Res. 2011;21:1863–71 21750105 10.1101/gr.116228.110 PMC3205571

[29] Hasson D, Panchenko T, Salimian KJ. et al. The octamer is the major form of CENP-A nucleosomes at human centromeres. Nat Struct Mol Biol. 2013;20:687–95 23644596 10.1038/nsmb.2562 PMC3760417

[30] Jedlicka P, Lexa M, Kejnovsky E. What can long terminal repeats tell us about the age of LTR retrotransposons, gene conversion and ectopic recombination? Front Plant Sci. 2020;11:644 32508870 10.3389/fpls.2020.00644 PMC7251063

[31] Chang X, He X, Li J. et al. High-quality *Gossypium hirsutum* and *Gossypium barbadense* genome assemblies reveal the landscape and evolution of centromeres. Plant Commun. 2024;5:100722 37742072 10.1016/j.xplc.2023.100722 PMC10873883

[32] Yi C, Liu Q, Huang Y. et al. Non-B-form DNA is associated with centromere stability in newly-formed polyploid wheat. Sci China Life Sci. 2024;67:1479–88 38639838 10.1007/s11427-023-2513-9

[33] Chen C, Wu S, Sun Y. et al. Three near-complete genome assemblies reveal substantial centromere dynamics from diploid to tetraploid in Brachypodium genus. Genome Biol. 2024;25:63 38439049 10.1186/s13059-024-03206-w PMC10910784

[34] Lv Y, Liu C, Li X. et al. A centromere map based on super pan-genome highlights the structure and function of rice centromeres. J Integr Plant Biol. 2024;66:196–207 38158885 10.1111/jipb.13607

[35] Bast J, Jaron KS, Schuseil D. et al. Asexual reproduction reduces transposable element load in experimental yeast populations. elife. 2019;8:e48548 31486772 10.7554/eLife.48548 PMC6783261

[36] Shi X, Cao S, Wang X. et al. The complete reference genome for grapevine (*Vitis vinifera* L.) genetics and breeding. Hortic Res. 2023;10:uhad061 37213686 10.1093/hr/uhad061 PMC10199708

[37] Zhang K, du M, Zhang H. et al. The haplotype-resolved T2T genome of teinturier cultivar Yan73 reveals the genetic basis of anthocyanin biosynthesis in grapes. Hortic Res. 2023;10:uhad205 38046853 10.1093/hr/uhad205 PMC10689054

[38] Wolfgruber TK, Sharma A, Schneider KL. et al. Maize centromere structure and evolution: sequence analysis of centromeres 2 and 5 reveals dynamic loci shaped primarily by retrotransposons. PLoS Genet. 2009;5:e1000743 19956743 10.1371/journal.pgen.1000743 PMC2776974

[39] Nagaki K, Talbert PB, Zhong CX. et al. Chromatin immunoprecipitation reveals that the 180-bp satellite repeat is the key functional DNA element of *Arabidopsis thaliana* centromeres. Genetics. 2003;163:1221–5 12663558 10.1093/genetics/163.3.1221 PMC1462492

[40] Liu Q, Yi C, Zhang Z. et al. Non-B-form DNA tends to form in centromeric regions and has undergone changes in polyploid oat subgenomes. Proc Natl Acad Sci USA. 2023;120:e2211683120 36574697 10.1073/pnas.2211683120 PMC9910436

[41] Liu C, Fu S, Yi C. et al. Unveiling the distinctive traits of functional rye centromeres: minisatellites, retrotransposons, and R-loop formation. Sci China Life Sci. 2024 10.1007/s11427-023-2524-0 38805064

[42] Ramani V, Deng X, Qiu R. et al. Massively multiplex singlecell Hi-C. Nat Methods. 2017;14:263–6 28135255 10.1038/nmeth.4155 PMC5330809

[43] Cheng H, Concepcion GT, Feng X. et al. Haplotype-resolved de novo assembly using phased assembly graphs with hifiasm. Nat Methods. 2021;18:170–5 33526886 10.1038/s41592-020-01056-5 PMC7961889

[44] Chen Y, Nie F, Xie SQ. et al. Efficient assembly of nanopore reads via highly accurate and intact error correction. Nat Commun. 2021;12:60 33397900 10.1038/s41467-020-20236-7 PMC7782737

[45] Vaser R, Sović I, Nagarajan N. et al. Fast and accurate de novo genome assembly from long uncorrected reads. Genome Res. 2017;27:737–46 28100585 10.1101/gr.214270.116 PMC5411768

[46] Walker BJ, Abeel T, Shea T. et al. Pilon: an integrated tool for comprehensive microbial variant detection and genome assembly improvement. PLoS One. 2014;9:e112963 25409509 10.1371/journal.pone.0112963 PMC4237348

[47] Chen S, Zhou Y, Chen Y. et al. Fastp: an ultra-fast all-in-one FASTQ preprocessor. Bioinformatics. 2018;34:i884–90 30423086 10.1093/bioinformatics/bty560 PMC6129281

[48] Zhang X, Zhang S, Zhao Q. et al. Assembly of allele-aware, chromosomal-scale autopolyploid genomes based on hi-C data. Nat Plants. 2019;5:833–45 31383970 10.1038/s41477-019-0487-8

[49] Durand NC, Robinson JT, Shamim MS. et al. Juicebox provides a visualization system for hi-C contact maps with unlimited zoom. Cell Syst. 2016;3:99–101 27467250 10.1016/j.cels.2015.07.012 PMC5596920

[50] Wolff J, Rabbani L, Gilsbach R. et al. Galaxy HiCExplorer 3: a web server for reproducible hi-C, capture hi-C and single-cell hi-C data analysis, quality control and visualization. Nucleic Acids Res. 2020;48:W177–84 32301980 10.1093/nar/gkaa220 PMC7319437

[51] Flynn JM, Hubley R, Goubert C. et al. RepeatModeler2 for automated genomic discovery of transposable element families. Proc Natl Acad Sci USA. 2020;117:9451–7 32300014 10.1073/pnas.1921046117 PMC7196820

[52] Xu Z, Wang H. LTR_FINDER: an efficient tool for the prediction of full-length LTR retrotransposons. Nucleic Acids Res. 2007;35:W265–8 17485477 10.1093/nar/gkm286 PMC1933203

[53] Ellinghaus D, Kurtz S, Willhoeft U. LTRharvest, an efficient and flexible software for de novo detection of LTR retrotransposons. BMC Bioinformatics. 2008;9:18 18194517 10.1186/1471-2105-9-18 PMC2253517

[54] Ou S, Jiang N. LTR_retriever: a highly accurate and sensitive program for identification of long terminal repeat retrotransposons. Plant Physiol. 2018;176:1410–22 29233850 10.1104/pp.17.01310 PMC5813529

[55] Abrusán G, Grundmann N, DeMester L. et al. TEclass—a tool for automated classification of unknown eukaryotic transposable elements. Bioinformatics. 2009;25:1329–30 19349283 10.1093/bioinformatics/btp084

[56] Stanke M, Diekhans M, Baertsch R. et al. Using native and syntenically mapped cDNA alignments to improve de novo gene finding. Bioinformatics. 2008;24:637–44 18218656 10.1093/bioinformatics/btn013

[57] Delcher AL, Bratke KA, Powers EC. et al. Identifying bacterial genes and endosymbiont DNA with glimmer. Bioinformatics. 2007;23:673–9 17237039 10.1093/bioinformatics/btm009 PMC2387122

[58] Manni M, Berkeley MR, Seppey M. et al. BUSCO update: novel and streamlined workflows along with broader and deeper phylogenetic coverage for scoring of eukaryotic, prokaryotic, and viral genomes. Mol Biol Evol. 2021;38:4647–54 34320186 10.1093/molbev/msab199 PMC8476166

[59] Kim D, Paggi JM, Park C. et al. Graph-based genome alignment and genotyping with HISAT2 and HISAT-genotype. Nat Biotechnol. 2019;37:907–15 31375807 10.1038/s41587-019-0201-4 PMC7605509

[60] Kovaka S, Zimin AV, Pertea GM. et al. Transcriptome assembly from long-read RNA-seq alignments with StringTie2. Genome Biol. 2019;20:278 31842956 10.1186/s13059-019-1910-1 PMC6912988

