# Supplementary material for: The gap-free genome of Forsythia suspensa illuminates the intricate landscape of centromeres
Source: Hortic Res. 2024 Jul 10;11(9):uhae185. doi: 10.1093/hr/uhae185 (PMC11374533; doi:10.1093/hr/uhae185)
Supplement: Web_Material_uhae185 [file web_material_uhae185.zip › supplementary_materials_uhae185.zip!/Supplemental Figures.docx]

**Figure S1. Gap reliability verification diagram.**

The bottom line represents the chromosome sequence, with the alignment of assembly reads/HiFi reads/ONT reads to this region shown above it. The dashed lines indicate the regions in the intermediate version of the genome that were replaced after gap filling.

**Figure S2. The workflow diagram of genome annotation analysis**

Figure S3: **Identification of centromeres using quarTeT.**

The mapped reads density was plotted in 100-kb windows along the fourteen chromosomes. The x-axis shows the positions on each chromosome. The y-axis represents ChIP/input ratio of the normalized density of uniquely mapped reads on each position, which was calculated based on the ratio of reads per million/100. Centromeric regions identified by the quarTeT are colored in green.

Figure S4: **Identification of repeat clusters enriched in *F. suspensa* centromeres**

A: Computational identification of repeat clusters that are enriched in *F. suspensa* centromeres. These repeat clusters are represented as dots on a scatter plot, where the x-axis shows the genomic abundance of the repeats and the y-axis is represented as the ratio of ChIP-seq reads to input reads for each repeat cluster. Only the top 50 most abundant repeat clusters, which are indicated by blue and red dots, are displayed in the scatter plot. Three repeat clusters marked with red dots were selected for cloning and confirmation by fluorescence *in situ* hybridization.

B-E: Snapshot of the distribution of the *F. suspensa* centromeric satellite repeat CentFs356 (B), CentFs530 (C), CentFs729 (D), and CentFs732 (E) occupied by CENH3 nucleosomes in the assembled Fsus-CHAU genome.

**Figure S5. Mapping of CENH3-ChIP-Seq in centromere 2 of *F. suspensa*.**

The first track illustrates CENH3 enrichment [log_2_(ChIP/Input)] plotted over *F. suspensa* centromere 2. The second and third tracks illustrate the distribution of CentFs356 and CentFs530, respectively, while the fourth and fifth tracks show the distribution of CentFs729 and CentFs732. Heatmaps display the pairwise sequence identity of *F. suspensa* centromeric satellites.

 **Figure S6. Phased arrangement of CENH3 nucleosomes on centromeric satellites.**

(A-D) The graph displays the distribution of midpoints for both the CENH3-ChIP-seq and input reads across the trimeric consensus sequence of the CentFs356 (A), CentFs365 (B), CentFs530 (C), and CentFs732 (D). The y-axis represents the read density, while the x-axis shows the position along the consensus sequence.
